# Influence of Tumor Immune Infiltration on Immune Checkpoint Inhibitor Therapeutic Efficacy: A Computational Retrospective Study

**DOI:** 10.3389/fimmu.2021.685370

**Published:** 2021-06-17

**Authors:** Rong Liu, Fang Yang, Ji-Ye Yin, Ying-Zi Liu, Wei Zhang, Hong-Hao Zhou

**Affiliations:** ^1^ Department of Clinical Pharmacology, Xiangya Hospital, Central South University, Changsha, China; ^2^ Hunan Key Laboratory of Pharmacogenetics, Institute of Clinical Pharmacology, Central South University, Changsha, China; ^3^ Engineering Research Center of Applied Technology of Pharmacogenomics, Ministry of Education, Changsha, China; ^4^ National Clinical Research Center for Geriatric Disorders, Changsha, China; ^5^ Department of Epidemiology and Health Statistics, Xiangya School of Public Health, Central South University, Changsha, China

**Keywords:** Tumour-immune infiltration, immune checkpoint inhibitor, response, progression-free survival, overall survival

## Abstract

The tumor immune microenvironment (TIME) is likely an important determinant of sensitivity to immune checkpoint inhibitor (ICI) treatment. However, a comprehensive analysis covering the complexity and diversity of the TIME and its influence on ICI therapeutic efficacy is still lacking. Data from 782 samples from 10 ICI clinical trials were collected. To infer the infiltration of 22 subsets of immune cells, CIBERSORTx was applied to the bulk tumor transcriptomes. The associations between each cell fraction and the response to ICI treatment, progression-free survival (PFS) and overall survival (OS) were evaluated, modeling cellular proportions as quartiles. Activity of the interferon-γ pathway, the cytolytic activity score and the MHC score were associated with good prognosis in melanoma. Of the immune cells investigated, M1 macrophages, activated memory CD4^+^ T cells, T follicular helper (Tfh) cells and CD8^+^ T cells correlated with response and prolonged PFS and OS, while resting memory CD4^+^ T cells was associated with unfavorable prognosis in melanoma and urothelial cancer. Consensus clustering revealed four immune subgroups with distinct responses to ICI therapy and survival patterns. The cluster with high proportions of infiltrated CD8^+^ T cells, activated memory CD4^+^ T cells, and Tfh cells and low levels of resting memory CD4^+^ T cells exhibited a higher tumor mutation burden and neoantigen load in melanoma and conferred a higher probability of response and improved survival. Local systemic immune cellular differences were associated with outcomes after ICI therapy. Further investigations of the tumor-infiltrating cellular immune response will lay the foundation for achieving durable efficacy.

## Introduction

Immune checkpoint inhibitors (ICIs), which modulate immune responses against tumors, have revolutionized the field of tumor therapy. ICIs work not only by blocking the receptor and/or ligand connections of molecules that participate in the inhibitory brakes of T cell activation or function but also by activating other cells to enhance the immune response against tumors. ICIs targeting cytotoxic T lymphocyte-associated protein 4 (CTLA-4; ipilimumab) and programmed cell death 1 (PD-1; pembrolizumab and nivolumab) and its ligand (programmed cell death-ligand 1, PD-L1; atezolizumab, durvalumab and avelumab) have been approved by the US Food and Drug Administration (FDA). In clinical practice, these antibodies are utilized as first-line medicines for a variety of solid and liquid tumors, such as melanoma (1), gastric cancer (2), and hematological malignancies (3), displaying an unprecedented prolongation of patient survival.

Although several patients have experienced dramatic tumor regression following ICI therapy across a variety of cancer indications, resistance to ICIs restricts the proportion of patients that can obtain a durable response. For example, for melanoma, one of the cancer types with the highest probability of sensitivity to ICIs, 60–70% of patients cannot achieve a clinical benefit from anti-PD-1 treatment; among those that do, 20–30% ultimately experience tumor progression (4). Generally, the mechanisms of resistance can be classified into two categories: primary and acquired. Patients who do not show an initial response to ICI therapy are referred to as having primary resistance or nonresponse; patients with acquired resistance are sensitive to ICIs in the beginning but develop resistance and experience tumor relapse or progression later. Dissecting the mechanisms of resistance should not only make it possible to predict a patient’s response to ICI therapy prior to treatment but ultimately could give some revelations for increasing the response rate. Therefore, great efforts have been made to dissect the mechanisms of resistance. Multiple factors have been revealed to influence ICI effectiveness, such as the PD-L1 expression level (4, 5), the mutational burden of the tumor (6, 7), and critical signaling pathways such as interferon-γ (IFN-γ) (8, 9). Nevertheless, as reviewed in the study by Bagchi et al., perhaps the most prominent predictive biomarker is the composition of tumor-infiltrating immune cells in patients before and after treatment with ICIs (10).

The tumor microenvironment mainly includes tumor cells and tumor-infiltrating immune cells admixed with stromal components, such as extracellular matrix and blood vessels. Studies on the tumor microenvironment have revealed the contributive roles of tumor-infiltrating immune cells in the therapeutic response and resistance to immunotherapy in various cancer types (11, 12). ICIs activate pre-existing tumor-infiltrating lymphocytes, which recognize and eliminate dysplastic and neoplastic cells, contributing to the response to immunotherapy (13). For instance, CD8^+^ T cells located in different positions and with different functional statuses have an effect on the response to ICIs. Intratumoral memory-like CD8^+^ T cells were positively correlated with the response to ICI treatment (14). Moreover, the existence of CD8^+^ T cells at the tumor invasive margins prior to the initiation of therapy was associated with a favorable ICI response, while the proliferation of intratumoral CD8^+^ T cells during therapy was correlated with tumor relapse or metastasis (15). Additionally, Sade-Feldman et al. conducted single-cell RNA-sequencing analysis on tumor biopsy specimens obtained at baseline from patients with advanced melanoma and demonstrated that patients who exhibited a response to ICIs had a larger number of memory CD4^+^ T cells (14). In addition to T cells, another cell type that has been studied extensively regarding ICI therapies is macrophages; for example, tumor-associated macrophages (M2 macrophages) display a variety of protumor effects by releasing immunosuppressive cytokines, such as transforming growth factor-b (TGF-b), that suppress antitumor immune responses and are associated with resistance to ICI treatment (16). The mechanisms by which tumor-infiltrating immune cells participate in the development of a systemic antitumor response are orchestrated or thwarted after ICI treatment are still under exploration, and comprehensive studies based on a large sample size that evaluate the relevance of immune cell fractions to patient prognosis after ICI therapy are urgently needed. However, so far, this type of research is still lacking.

As technology has advanced and there are growing numbers of ICI trials with available omics data, it is possible for us to conduct large-scale analysis with cohorts covering the multiformity of the immune context of the TIME, which influences the response to ICI therapy. A computational framework, CIBERSORTx (17), which accurately estimates the relative proportions of 22 immune cell types with distinct functions, was applied to 782 tumor transcriptomes from 10 publicly available ICI-related datasets. We observed tumoral and systematic immune cell milieus before ICI therapy and tested the degree of correlation with response, PFS and OS, across cancers (pan cancer) and by cancer types, targets and drugs. A deeper understanding of the diversity of the association between ICI efficacy phenotypes and different functional immune cell subsets was obtained, and this information was used to predict and guide immunotherapeutic responsiveness.

## Methods and Materials

### Gene Expression Data of Patients Treated With Immunotherapy and Data Processing

**Figure 1:**
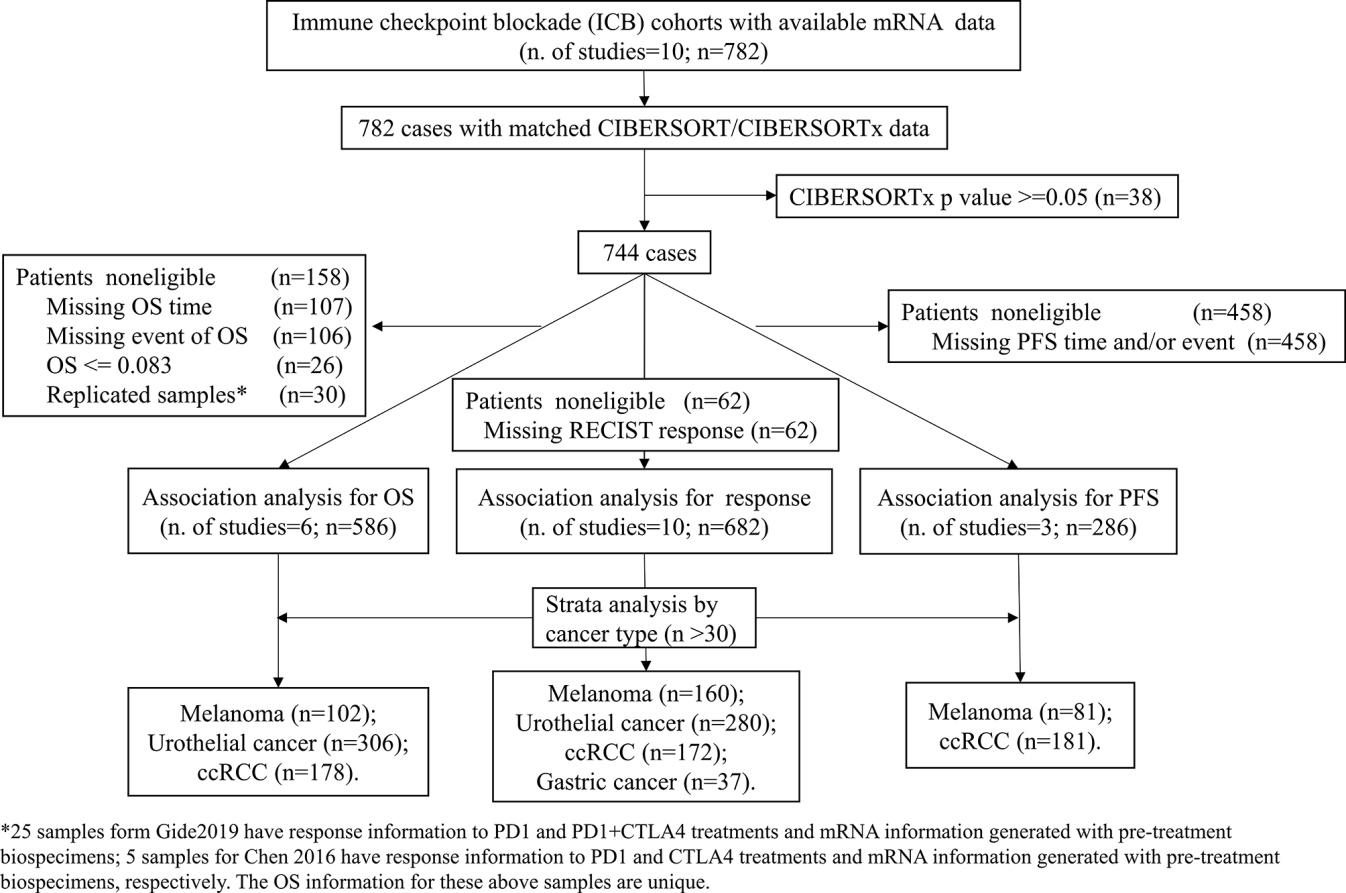
Study flow diagram. OS, Overall survival; PFS, Progression-free survival, ccRCC, clear cell renal cell carcinoma.

This computational retrospective study takes advantage of ICI trials with publicly available omics data. The clinical and matched mRNA expression data of tumors from 10 published ICI clinical studies (listed in Additional file 1: **Table S1**) were collected. Datasets were labeled as the first author plus the publication year. For the Chen 2016 (18), Hugo 2016 (19), Prat 2017 (20), Nathanson 2017 (21), Riaz 2017 (22), Kim 2018 (23), and Gide 2019 (24) datasets, the processed mRNA datasets were downloaded from the TIDE data portal (http://tide.dfci.harvard.edu/download/) on November 23, 2020. As described in the study by Fu et al., for each RNA-seq dataset, the transcriptomic profiles were log2 (1+ transcripts per kilobase million [TPM]) transformed, the log scale transcriptome data across patients were standardized by quantile normalization, and then the expression values of each gene were further normalized by subtracting the average among all samples. Only samples from ICI pretreatment tumors were included. For the Braun 2020 (25), the mRNA and clinical datasets were download from the supplementary of the original paper. The Mariathasan 2018 (26) dataset was obtained from the R package “IMvigor210CoreBiologies” (http://research-pub.gene.com/IMvigor210CoreBiologies). To be consistent with the above datasets, we transformed the count values into log2(TPM+1), standardized the log-transformed values across patients by quantile normalization, and further normalized the expression values of each gene by subtracting the mean value among all samples. The processed expression matrix was utilized for downstream analyses. The supplementary files of the original publication were searched to collect clinical information. **Figure 1** illustrates the samples used at each phase of the statistical analysis.

### Immune Response–Related Signatures

Herein, four kinds of immune response–related gene signatures were assessed. To evaluate the immune score that measured the content of tumor-infiltrating immune cells in each patient across different datasets, ESTIMATE (Estimation of STromal and Immune cells in MAlignant Tumor tissues using Expression data) was utilized (27). The MHC score was calculated as the average expression level of the “core” MHC-I gene set, which contains NLRC5, TAP1, TAP2, HLA-A, HLA-B, HLA-C, PSMB9, PSMB8, and B2M (28). The cytolytic activity score (CYT score) was formulated according to the average expression levels of the granzyme A (GZMA) and perforin (PRF1) genes defined in a previous study (29). Gene lists of the IFN-γ pathway were accessed from the Molecular Signatures Database (30). The pathway score was calculated as the mean expression levels of the genes included in this pathway.

### Infiltrating Immune Cell Subsets

To estimate the proportions of immune cell subsets in a mixed cell population from RNA-seq data, the CIBERSORTx (17) (https://cibersortx.stanford.edu/) deconvolution algorithm was used (Submitted job type: ‘Impute cell fractions’). The LM22 signature was set as a reference, and the relative immune cell proportions from each processed dataset were estimated in 1000 permutations with B mode batch correction, with quantile normalization disabled for RNA-seq data. The B mode batch correction mode was utilized to correct for batch effects between the LM22 signature generated from the microarray-based dataset and the dataset utilized here, which consisted of RNA-seq data. A p value that measures the reliability of the deconvolution results was computed, and samples with a p value >0.05 were discarded from downstream association analyses. For the Uppaluri 2020 dataset (31), the cell proportions in absolute mode generated with CIBERSORT, the former version of CIBERSORTx, rather than normalized RNA-seq data were provided. Relative cell fractions were obtained by normalizing the CIBERSORT output to the sample-level total number of immune cell counts.

### Clinical Outcomes

Clinical information on age, sex, ICI targets, ICI drugs, response to ICI treatment and survival was collected. Tumor response was determined using the Response Evaluation Criteria in Solid Tumors (RECIST) as described in the original publications. We categorized patients with complete response (CR) and partial response (PR) as responders and compared them with non-responders who displayed stable disease (SD) or progressive disease (PD). Samples with CR, PR, and SD > 6 months were considered to show clinical benefit, while patients with SD <= 6 months and PD were classified as nonclinical benefit. The clinical endpoints were response, clinical benefit, PFS and OS. The clinical outcomes provided in each dataset are listed in **Table S1**.

### Statistical Analysis

To test the association between the inferred fractions of immune cell types and response or clinical benefit, logistic regression analysis was performed. Patients with a CIBERSORTx p-value of ≥0.05 were filtered. To derive more easily interpretable ORs, in the logistic regression models, quartiles (25%, 50% and 75%) of the proportions of each immune cell type were modeled as continuous variables. To evaluate the associations between the immune cell types in the tumor microenvironment and progression-free survival (PFS) and overall survival (OS), survival analysis was conducted. Patients with a follow-up time or OS time of less than 1 month who were likely to have postoperative complications were excluded. Cox regression analyses were stratified by study, with quartiles of each of the 22 immune cell subsets modeled as a continuous variable. The Kaplan-Meier method was used to draw survival curves for the subgroups. The log rank test was used to evaluate the difference in the survival rates. We also tested the associations between clinical outcomes and age and grade. Covariates and immune cell subtypes significantly associated with outcomes in univariate analyses were included in the multivariable models.

Furthermore, to address the possibility that choosing variables from univariate analysis may result in the exclusion of some associated variables when confounding is properly controlled (32), multivariable logistic and Cox regression models were fitted (with 22 immune cell subsets as predictors accounting for the possibility of confounding) *via* the penalized maximum likelihood using the “glmnet” package (33) for dichotomous dependent variables and for time-to-event data. The penalization factor was selected according to 1,000 rounds of cross-validation.

To explore whether distinct patterns of tumor-infiltrating immune cells were represented, we performed consensus clustering analysis on the immune cell subsets of cases with a CIBERSORTx p value less than 0.05. To ensure comparability between the low and high overall proportion of cell subtypes, the values of relative immune cell fractions were rescaled between zero and one for each cell subset. The unsupervised clustering “Pam” algorithm based on Manhattan distance was chosen, executed by using the “ConsensusClusterPlus” R package (34), with 1,000 repeats for stable classification.

Pairwise correlations between immune cell subtypes were assessed with Pearson’s correlation analysis and depicted in heatmaps using the “pheatmap” R package. The chi-square test was used to analyze the correlations between categorical variables. The associations between the quantiles of the immune response signature and clinical outcomes were tested with the abovementioned statistical methods. The association between immune clusters and clinical outcomes was tested with the chi-square test for response and survival analysis with PFS or OS. The difference in mutation burden and neoantigen load between immune clusters was evaluated with one-way ANOVA.

All statistical analyses were performed with R software version 3.6.3 (35). The cutoff for a statistically significant p value was 0.05.

## Results

### Response to ICI Therapy of the Study Populations

**Figure 2:**
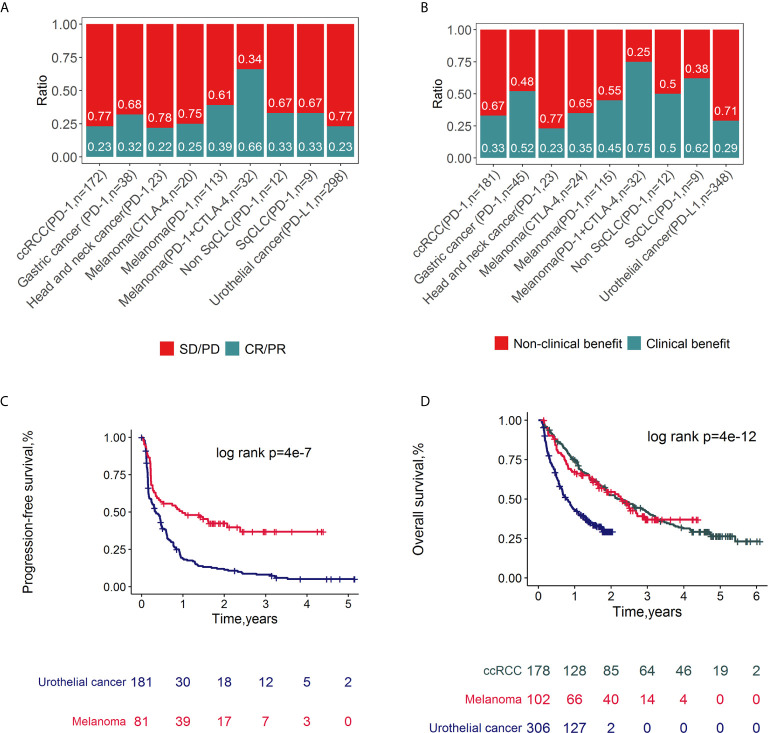
Clinical outcomes of ICI related datasets. Spine plots for response **(A)** and clinical benefit **(B)** and survival plots for PFS **(C)** and OS **(D)** and by cancer types. P-values from log-rank tests are depicted.

Classical clinicopathologic factors (age, sex, grade, drug, target, clinical benefit, response, OS and PFS) were collected (**Tables S1, S2**). The ratios of response (**Figure 2A**) and clinical benefit (**Figure 2B**) to different ICI therapy targets (PD-1, PD-L1 and CTLA-4) across cancer subtypes were calculated and compared. Since the response and clinical benefit concordantly overlapped (**Figure S1**), response was selected as one of the main clinical outcomes in the following association analysis. Overall, the response ratios of melanoma to ICI therapy (combined with PD-1 and CTLA-4 inhibitors) were 66%, which was the highest among these ICI trails. Meanwhile, melanoma patients treated with CTLA-4 inhibitors had a low response ratio (25%). The cancer type with the lowest response ratio to ICI therapy among these cancers was head and neck cancer, with a ratio of 22%. The PFS (**Figure 2C**) and OS (**Figure 2D**) rates across cancer subtypes treated with ICIs were compared and depicted. Patients with melanoma represented a higher PFS and OS rates compared with urothelial cancer (log rank p<0.05). There is no significant difference between the OS rates of melanoma and clear cell renal cell carcinoma (ccRCC).

### Leukocyte Composition in Bulk Tumors

**Figure 3:**
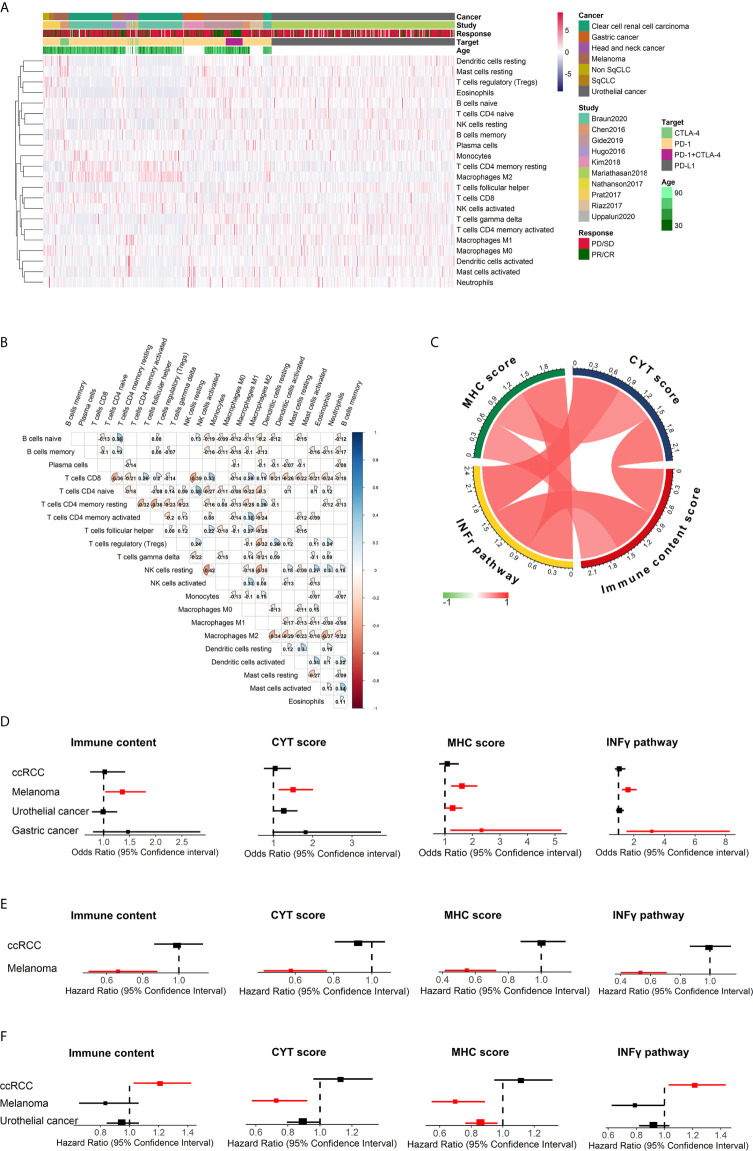
Summary of the estimated relative fractions of 22 tumor-associated leukocytes in this study and immune response prediction signatures in melanoma. **(A)** Heatmap of unsupervised clustering of tumor-infiltrating immune cells in cohorts treated with ICIs. Rows and columns represent tumor-infiltrating immune cells and samples, respectively. **(B)** Heatmap of the correlation coefficient matrix of all 22 immune cell proportions across cancers. **(C)** correlation of immune scores. Forest plot demonstrating ORs (boxes) and 95% CIs (horizontal lines) for the association with response to ICI therapy for these four immune signatures **(D)**. Forest plot demonstrating HRs (boxes) and 95% CIs (horizontal lines) for the association with PFS **(E)** and OS **(F)** to ICI therapy for these four immune signatures. In the forest plots, significant associations were colored with red. NK cells, natural killer cells; sqCLC, squamous cell lung carcinoma; Non-SqCLC, non–small cell lung carcinoma; SD, stable disease; PD, progressive disease; PR, partial response; CR, complete response.

The individual relative infiltration levels of 22 distinct leukocyte subsets were deconvoluted from the transcriptome data of 782 patients in ICI clinical cohorts and summarized (**Figure 3A**). The ratios of 547 genes composing the LM22 signature that were available for CIBERSORTx analysis in each study are depicted in **Figure S2**. The average number of genes represented was 77.8%. A total of 95.1% of samples (744/782) had CIBERSORTx p < 0.05. Among these patients, the least and most variable immune cell types were activated NK cells (1.54% ± 2.23%) and M2 macrophages (11.25% ± 10.82%), respectively. All 22 immune cell subsets showed infiltration in over 50% of the samples **(** **Figure S3** **).**

The fractions of the 22 immune cell subsets were weakly to moderately correlated with cancers overall (**Figure 3B**) and with melanoma (**Figure S4A**), urothelial cancer (**Figure S4B**), gastric cancer (**Figure S4C**) and ccRCC (**Figure S4D**). Generally, naïve CD^4+^ T cells and resting NK cells showed the strongest positive correlation (R=0.38), whereas activated NK cells and resting NK cells showed the strongest negative correlation (R=-0.42).

### Transcriptome-Based Immune Response Score

Gene signatures have been shown to affect the immune response of patients. The associations between clinical outcomes after ICI therapy and the following four distinct immune gene expression signatures were evaluated: (1) immune score, which is a measure of the total immune infiltration in the tumor; (2) IFN-γ pathway activity, which is a critical cytokine for activating and sustaining an effective antitumor effect (8); (3) cytolytic activity, which is a measure of cytolytic enzymes used by immune cells to kill tumor cells; and (4) MHC score, which is a measure of antigen presentation required for tumor cell recognition by T cells and subsequent T cell–mediated killing. These four immune-related signatures were calculated for all cancer patients included in this study, and positive correlations were found between them (**Figure 3C**). Then, the association between the immune signatures and clinical outcomes was investigated across cancer types, and we found diversity across cancer types (**Figures 3D–F**). For melanoma, patients with higher immune content (odds ratio [OR] 1.36, 95% CI 1.03-1.81, p=0.032), CYT scores (OR 1.50, 95% CI 1.13-2.01, p=5.34×10^-3^) MHC scores (OR 1.62, 95% CI 1.22-2.18, p=9.90×10^-4^) and IFN-γ scores (OR 1.62, 95% CI 1.22-2.18, p=1.12×10^-3^) were associated with an increased probability of response to immunotherapy and significantly improved PFS (p<0.05). The associations between immune content, IFN-γ and OS were not significant for melanoma (**Figure 3F**). Gastric cancer patients with higher IFN-γ (OR 3.17, 95% CI 1.51-8.26, p=6.24×10^-3^) and MHC (OR 2.33, 95% CI 1.19-5.23, p=0.02) scores tended to have a higher probability of response to ICIs. However, for ccRCC, patients with higher IFN-γ signature associated with unfavorable OS after ICI therapy (HR=1.22, 95% CI 1.03-1.43, p=0.022).

### Immune Predictors of Response to ICI Therapy

**Figure 4:**
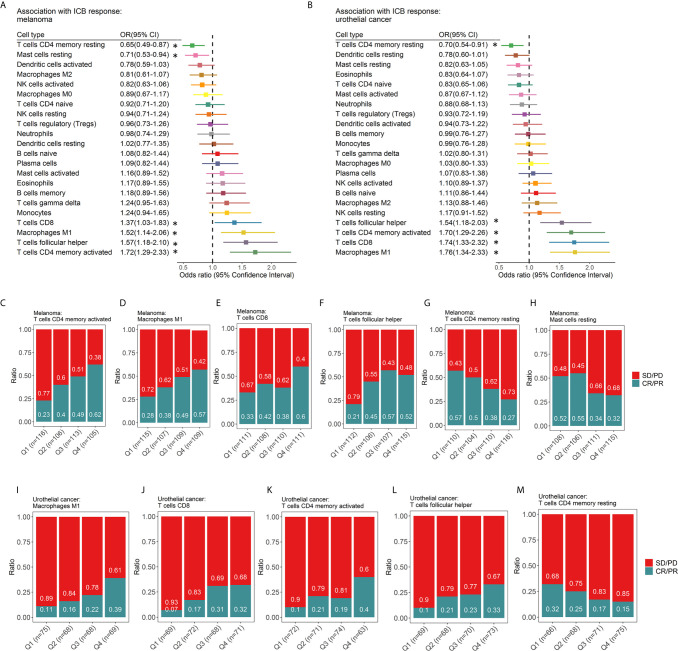
Associations between ICI response and tumor-infiltrating immune cell subtypes. Forest plot demonstrating ORs (boxes) and 95% CIs (horizontal lines) for the association with response to ICI therapy in melanoma **(A)** and urothelial cancer **(B)**. The size of the box is negatively proportional to the standard error of the OR. * denote ORs with a p-value < 0.05. Spine plots demonstrating the distribution of response rates within quartiles of immune cell subsets **(C-M)**. OR, odds ratio; CI, confidence interval.

The relationship between immune cell subsets and response to ICIs was evaluated (**Figure 4**). We found that patients who achieved CR/PR had prolonged survival for melanoma (HR 0.04, 95% CI 0.02-0.10 p=2.30×10^-11^ for PFS, **Figure S5A**; HR 0.05, 95% CI 0.02-0.13 p=2.21×10^-9^ for OS, **Figure S5B**), ccRCC (HR 0.05, 95% CI 0.11-0.27, p=7.0×10^-16^ for PFS, **Figure S5C**; HR 0.05, 95% CI 0.03-0.09, p<2.0×10^-16^ for OS, **Figure S5D**) and urothelial cancer (HR 0.04, 95% CI 0.02-0.10, p= 2.68×10^-12^ for OS, **Figure S5E**). For melanoma (**Figure 4A**), activated memory CD4^+^ T cells (OR 1.72, 95% CI 1.29-2.33; p=2.72×10^-4^, **Figure 4C**), M1 macrophages (OR 1.52, 95% CI 1.14-2.06; p =4.77×10^-3^, **Figure 4D**), CD8^+^ T cells (OR 1.37, 95% CI 1.03-1.83; p =2.98×10^-2^, **Figure 4E**), Tfh cells (OR 1.57, 95% CI 1.18-2.10; p =2.20×10^-3^, **Figure 4F**) were significantly associated with response, while resting memory CD4^+^ T cells (OR 0.65, 95% CI 0.49-0.87; p=3.62×10^-3^, **Figure 4G**) and resting mast cells (OR 0.71, 95% CI 0.53-0.94; p=1.78×10^-2^, **Figure 4H**) showed a significant association with a lack of response and, therefore, resistance to ICI therapy. For urothelial cancer (**Figure 4B**), M1 macrophages (OR 1.76, 95% CI 1.34-2.33; p =5.69×10^-5^, **Figure 4I**), CD8^+^ T cells (OR 1.74, 95% CI 1.33-2.32; p =8.55×10^-5^, **Figure 4J**), activated memory CD4^+^ T cells (OR 1.70, 95% CI 1.29-2.26; p=1.95×10^-4^, **Figure 4K**), and Tfh cells (OR 1.54, 95% CI 1.18-2.03; p =1.60×10^-3^, **Figure 4L**) were significantly associated with response, while resting memory CD4^+^ T cells (OR 0.70, 95% CI 0.54-0.91; p=8.03×10^-3^, **Figure 4M**) showed a significant association with a lack of response. In gastric cancer, M1 macrophages, CD8^+^ T cells, and activated natural killer (NK) cells were associated with an increased probability of response, while resting CD4^+^ T cells, naïve CD4^+^ T cells, naïve B cells and activated mast cells were correlated with a decreased probability of response (**Figure S6**). Due to the relatively small sample size (n=37) of gastric cancer, these findings still need further validation. However, no relationships between immune cells and response to ICI were statistically significant for ccRCC (**Figure S6B**). The relationship between immune cell subsets and clinical benefit following ICI therapy was also evaluated, and the results were in accordance with those of response (**Figure S7** **).** Univariate and multivariate analyses were conducted for melanoma (**Table S3**) and urothelial cancer (**Table S4**). In melanoma, univariate analysis revealed that age and sex were not associated with the response to ICI therapy (**Table S3A**). Multivariate analysis (**Table S3B**) revealed that activated memory CD4^+^ T cells (OR 1.59, 95% CI 1.17-2.20; p=3.83×10^-3^) contributed to the adjusted model. In multivariable logistic regression models with penalized maximum likelihood estimation, activated memory CD4^+^ T cells (OR=1.54) and Tfh cells (OR=1.36) demonstrated high OR point estimates (**Table S3C**). Activated memory CD4^+^ T cells (OR 1.47, 95% CI 1.08-2.04; p=1.76×10^-2^) and Tfh cells (OR=1.46, 95% CI 1.08-1.99, p=1.45×10^-2^) contributed to the adjusted model for urothelial cancer.

### Prognostic Associations of Tumor-Infiltrating Leukocytes

**Figure 5:**
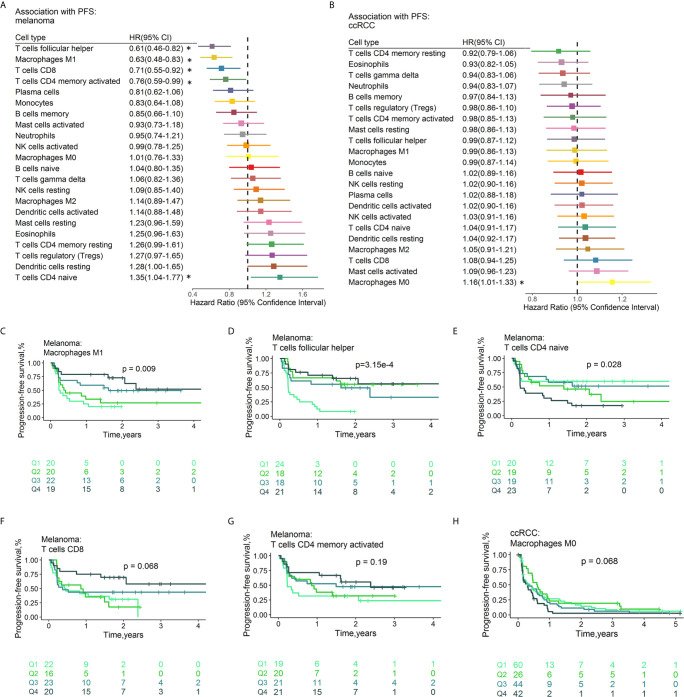
Associations between PFS and immune cell subtypes. Forest plot demonstrating HRs (boxes) and 95% CIs (horizontal lines) for the association with PFS after ICI therapy in melanoma **(A)** and ccRCC **(B)**. The results were obtained from univariate Cox regression analysis. The size of the box is negatively proportional to the standard error of the HR. * denote HRs with a p-value < 0.05. Survival curves of quartiles of immune cell fractions **(C–H)**. Illustrated p-values are from log-rank tests. HR, hazard ratio, OS, Overall survival, CI, confidence interval.

**Figure 6:**
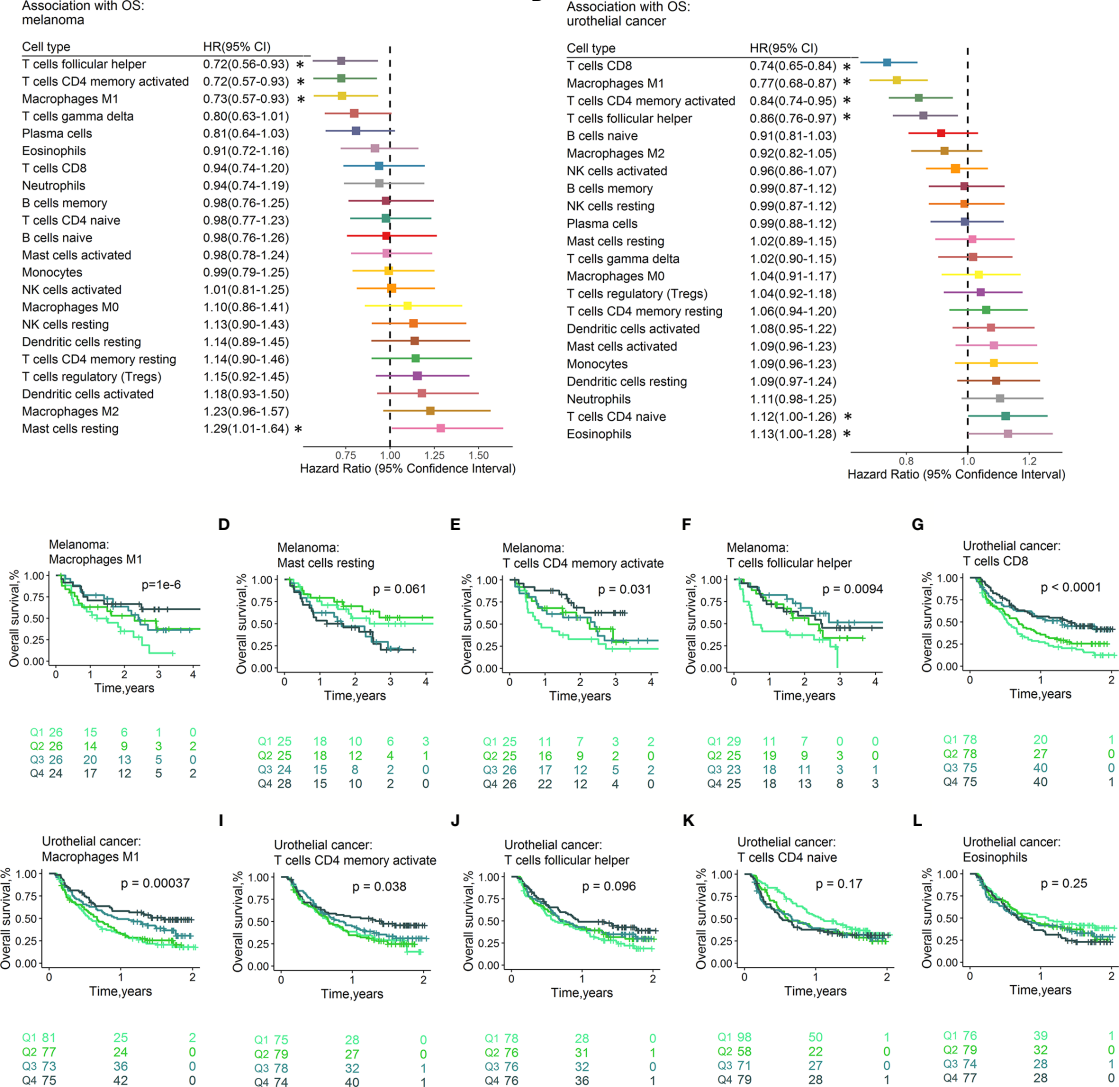
Associations between OS and immune cell subtypes. Forest plot demonstrating HRs (boxes) and 95% CIs (horizontal lines) for the association with PFS after ICI therapy in melanoma **(A)** and urothelial cancer **(B)**. The results were obtained from univariate Cox regression analysis. The size of the box is negatively proportional to the standard error of the HR. * denote HRs with a p-value < 0.05. Survival curves of quartiles of immune cell fractions **(C–L)**. Illustrated p-values are from log-rank tests. HR: hazard ratio. PFS, Progression-free survival; CI, confidence interval.

The fractions of tumor-infiltrating immune cells were correlated with the survival of tumor patients treated with ICIs. A total of 286 patients had RFS information (median RFS time =0.40 years, 223 with disease progression). A total of 586 patients remained after removing those with OS ≤ 1 month, with a median OS time of 1.17 years (379 events). The HRs and 95% CIs of immune cell subtypes for PFS (**Figure 5**) and OS (**Figure 6** **)** are illustrated with forest plots. Association analyses to evaluate the prognostic effect of immune cells by cancer type (n>10) were performed. There were variations in terms of the prognostic effect of immune cells by cancer type. In ccRCC, M0 macrophages were significantly associated with poor PFS (HR=1.16, 95% CI=1.00–1.33; p=4.23×10^-2^) and OS (HR=1.24, 95% CI=1.06–1.44; p=6.77×10^-3^) (**Figure S8**). In melanoma, three types of immune cells were significantly associated with the survival of patients after treatment with ICI drugs: Tfh cells (HR 0.61, 95% CI 0.46-0.82, p=8.56×10^-4^ for PFS; HR 0.72, 95% CI 0.56-0.93, p=1.22×10^-2^ for OS), M1 macrophages (HR 0.63, 95% CI 0.48-0.83, p=1.05×10^-3^ for PFS,; HR 0.73, 95% CI 0.57-0.93, p=1.21×10^-2^ for OS), and activated memory CD4^+^ T cells (HR 0.76, 95% CI 0.59-0.99, p=3.82×10^-2^ for PFS; HR 0.72, 95% CI 0.57-0.93, p=1.01×10^-2^ for OS). These findings might somewhat account for the association between these immune cell subsets and a favorable response to ICI therapy. Moreover, naïve CD4^+^ T cells were associated with poor PFS (HR=1.35, 95% CI=1.04-1.77, p=2.68×10^-2^). Neither age nor sex was associated with PFS (**Table S3D**) or OS (**Table S3E**). Multivariable Cox regression analyses were conducted, and the results revealed that none of these immune subsets left in the model for PFS (**Table S4F**) and OS (**Table S4G**) may accounting for the relatively small sample size. In multivariable Cox regression models with penalized maximum likelihood estimation, the immune cells with the lowest HR estimates for PFS (**Table S3H**) and OS (**Table S3I**) were follicular helper T cells (HR=0.82) and M1 macrophages (HR=0.87), respectively. In urothelial cancer, CD8+ T cells (HR =0.80, 95% CI=0.69-0.93; p=3.53×10^-3^) contributed to the multivariate model (**Table S4E**) and with the lowest HR estimate (HR=0.86) in multivariable Cox regression models with penalized maximum likelihood estimation for OS (**Table 4F**).

### Identified Immune Clusters

To explore whether different patterns of the TIME could be distinguished based on the 22 immune cell subsets, consensus clustering (34) of all samples was conducted.

**Figure 7:**
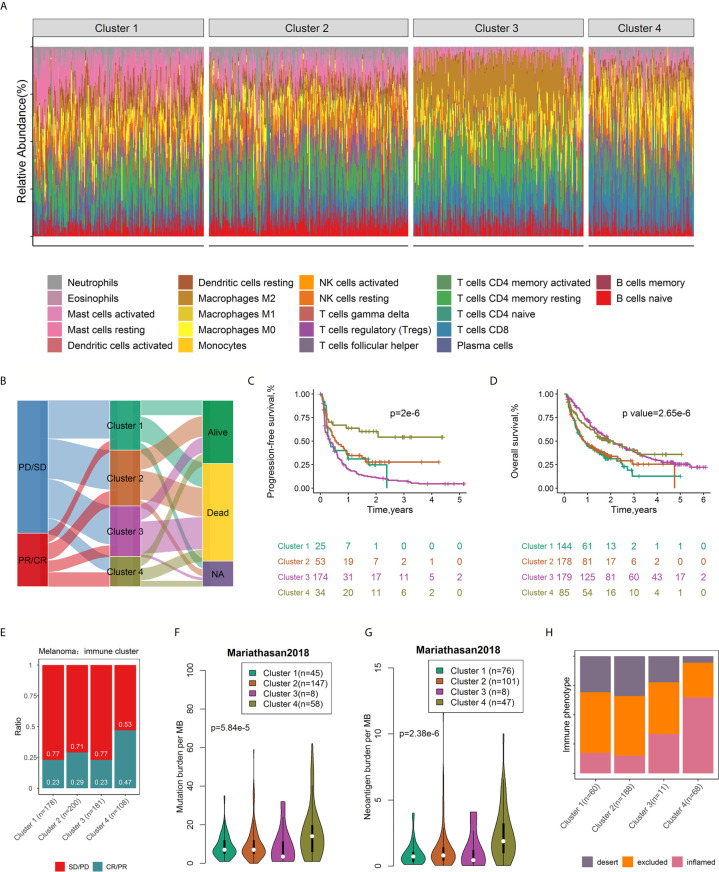
Consensus clustering of 782 samples based on immune cell ratios. Stacked bar charts of immune cell proportions by immune cluster **(A)**. Alluvial diagram of immune cluster distribution in patients with different immune clusters, responses to ICI therapy, and OS outcomes **(B)**. Survival plots by immune cluster separately for OS **(C)** and PFS **(D)**. P-values calculated with log-rank tests are represented. Spine plots depicting the correlation between immune clusters and response in melanoma **(E)**. Differences in mutation burden **(F)** and neoantigen burden **(G)** among distinct immune clusters (ANOVA test, p < 0.0001). Spine plots depicting the correlation between immune clusters and immune phenotype **(H)**. OS, overall survival; PFS, progression-free survival.

We assessed the cumulative distribution function, the proportion of ambiguous cluster values, and the consensus matrix and consensus cluster index, which indicated four clusters in the data (**Figure S9**). The tumor-infiltrating immune cell ratios by cluster are presented in **Figure 7A**, and their distributions are shown as box plots in **Figure S10**. The distribution of patients in the four immune clusters is illustrated in **Figure 7B**. Survival analysis revealed differences in RFS (log rank p=2.0×10^-6^, **Figure 7C**) and OS (log rank p=2.65×10^-6^, **Figure 7D**) among the immune subtypes. Distinct probabilities of response to ICI therapy (p value=1.79×10^-3^, **Figure 7E**) were shown between immune clusters. Cluster 4, characterized by high proportions of CD8^+^ T cells, activated memory CD4^+^ T cells, and Tfh cells and low levels of resting memory CD4^+^ T cells, conferred a higher probability of response (47% *vs* 26% on average) and improved RFS and OS after ICI therapy.

### Correlation Between the Immune Clusters and Mutation Load

Studies have shown that tumor mutation burden (TMB, nonsynonymous variants) might determine patients’ response to ICI therapy. A higher TMB level was positively correlated with the probability of response to PD-1 inhibitors and favorable PFS in the KEYNOTE 012 clinical trial (6, 36). In addition, tumor neoantigen burden (TNB) was positively associated with response to atezolizumab and OS (26). Taking the critical clinical implications of TMB and TNB into consideration, we investigated the intrinsic correlation between TMB/TNB and immune clusters. Notably, using the Mariathasan2018 dataset with mutation load and neoantigen load information available for each sample, we compared the TMB and neoantigen load burden within different immune clusters and found a significant association between immune cluster and mutation load (p= 3.41×10^-8^, **Figure 7F**), neoantigen load (p= 2.52×10^-13^, **Figure 7G**) and immune phenotype (p<2.2×10^-16^, **Figure 7H**). Patients in cluster 4 exhibited a significantly higher TMB and neoantigen load than those in the other clusters. Immune clusters were significantly associated with the immune phenotype (desert, excluded and inflamed), largely due to the enrichment of the inflamed subtype (64.81% to 26.12% overall) and the lack of the desert subtype (17.31% to 27.23% overall) in cluster 4 (**Figure 7H**). Infiltrated–inflamed tumor immune microenvironments, characterized by high tumor-infiltrating cytotoxic lymphocytes expressing PD-1 and leukocytes and tumor cells expressing PD-L1, are considered to be immunologically ‘hot’ tumors that exhibit an effective response to ICIs (11). Collectively, these findings suggest that the diversity of tumor-infiltrating immune cells to some degree attributed to the molecular features of the tumor.

## Discussion

The relationship between tumor-infiltrating immune cells and the antitumor effects of ICIs is complicated. By using CIBERSORTx, a computational technique that can accurately estimate leukocyte constituents in bulk tumors, the distinct patterns of immune cell infiltration across tumors and the complicated relationships with response, RFS and OS of 22 immune cell fractions based on different cancer types, targets and drugs were revealed. Our findings are in line with those of the limited literature, which suggests the reliability of our findings.

Previous studies have shown that the immune content score, the MHC score, the CYT score and IFN-γ pathway activity are associated with the immune response. For instance, cytolytic activity was measured across eighteen kinds of tumor types, and higher expression was found to be correlated with counterregulatory immune responses and favorable prognosis (29). IFN-γ, as an important cytokine in antitumor immunity, works by increasing cytotoxic CD8^+^ T cell activity, contributing to a Th1 response, exerting antiproliferative effects and so on (8). A highly activated IFN-γ pathway has been proposed as an effective predictor of patients’ response to anti-PD-1 agents (4, 9). In addition, MHC molecules participate in the development of the immune response. In this retrospective study, we made full use of publicly available ICI datasets and found that higher levels of these four scores were associated with an increased probability of response to ICI therapy in melanoma and gastric cancer and favorable PFS and OS in melanoma. However, these associations were not found in ccRCC, reflecting the exhausted phenotype (37).

Computational techniques, such as CIBERSORTx (17), xCell (38) and TRUST (39), were established for the estimation of cellular heterogeneity from the transcriptome data of mixed samples. Using CIBERSORTx (17) to infer the relative ratios of 22 leukocyte fractions from gene expression profiles, we have performed, as far as we know, the most comprehensive analysis of the prognostic impact of tumor-associated leukocytes in patients treated with ICIs until now. Some immune suppressor cells (CD8^+^ T cells, Tfh cells, activated memory CD4^+^ T cells and M1 macrophages) were found to be correlated with favorable clinical outcomes, but resting memory CD4^+^ T cells and resting mast cells showed negative associations with prognosis across cancers and in melanoma and urothelial cancer. Traditionally, CD8^+^ T cells have been implicated as important effector cells responsible for the induction of antitumor immunity. Studies have demonstrated that the increased amount of memory-like CD8^+^ T cells either in peripheral tissues or infiltrating tumor tissues before or after the initiation of treatment is predictive of a favorable response to ICIs (14, 15, 40, 41). Kilian and colleagues found that the abundance of CD8^+^ effector memory cells in the blood circulation of patients with metastatic melanoma was positively associated with the response to ICIs (40). It should be noted that the functional status of CD8+ T cells within tumors have been associated with diverse ICI therapy outcomes. For instance, CD45RA−CD8+ T cells and CD8+ T effector memory cells in the peripheral blood of melanoma at baseline were positively associated with response to ipilimumab treatment, while CD45RA+ naive CD8+ T cells were more abundant in non-responsive patients (41). Subrahmanyam et al. (41) showed that CD4^+^ and CD8^+^ memory T cell subsets in the peripheral blood of melanoma patients prior to therapy play critical roles in the response to anti-CTLA-4. However, although not statistically significant (OR=0.76, 95% CI=0.54-1.05, p=9.67×10^-2^ for response; HR=1.09, 95% CI=0.93-1.27, p=3.06×10^-1^ for OS), a higher number of infiltrated CD8^+^ T cells in ccRCC was associated with poor prognosis, which is in line with the findings of previous studies (37, 42). Similar to CD8^+^ T cells, it has been suggested that the abundance of CD4^+^ T cell subsets prior to treatment has predictive ability in distinguishing responders from nonresponders to ICIs. Evidence suggests that CD4+ T cells play a potential role in the antitumor immune response. It has been reported that a higher abundance of CD4^+^ T cells in blood is associated with prolonged OS in melanoma patients treated with ipilimumab (135). In a recent study, Kagamu et al. (43) showed that a heightened frequency of CD62LlowCD4^+^ T cells in NSCLC patients’ peripheral blood at baseline was predictive of favorable PFS following anti-PD-1 treatment. Similarly, single-cell RNA-sequencing analysis conducted on advanced melanoma biopsies showed that ICI responders had a higher content of memory CD4^+^ T cells at baseline (14). In our study, we found that a high level of activated memory CD4^+^ T cells and a low level of resting memory CD4^+^ T cells were correlated with a higher response rate and prolonged survival of ICI-treated melanoma patients.

Tfh cells, as specialized T helper cells, release the chemokine CXC-chemokine ligand 13 (CXCL13), which targets B cells and Tfh cells themselves through CXC-chemokine receptor 5. Both a high abundance of Tfh cells and a high expression level of CXCL13 correlated with increased survival in breast cancer (44) and colorectal cancer (45). In a recent study using a mouse model of triple-negative breast cancer in a preclinical trial of anti-CTLA-4 and anti-PD1 therapy, Daniel et al. found that B cells and Tfh cells were direct mediators of the ICI response (46). Tfh cells have not been deeply explored in terms of the response to ICI therapy, which needs further exploration. Overall, we found that high fractions of M0 macrophages were associated with poor survival in ccRCC, while high fractions of M1 macrophages conferred favorable prognosis, regardless of response, RFS and OS, in melanoma and urothelial cancer. Macrophages are tissue-resident differentiated monocytes with phagocytic activity. M0 macrophages can be polarized into M1 and M2 subtypes, which display distinct immunoregulatory characteristics. M1 macrophages have proinflammatory properties and promote antitumor TH1-type responses, whereas M2 macrophages are anti-inflammatory in nature and show protumoral activity. This study emphasizes the prognostic effect of M1 macrophages on ICIs. However, the mechanism by which M1 macrophages contribute to the immune response to ICIs still needs further experimental exploration. This study highlights the necessity of taking the variability of macrophage functional subtypes into full consideration.

The consensus clustering analysis based on tumor-infiltrating immune cells displayed four immune clusters with distinct prognoses. In addition, immune clusters were correlated with tumor TMB, TNB and immune phenotypes. Cluster 4, featuring high levels of CD8^+^ T cells, Tfh cells, M1 macrophages, and activated CD4^+^ T cells and low levels of resting CD4^+^ T cells, had high TMB and TNB levels. TMB is a well-recognized biomarker, and higher tumor mutational loads are closely correlated with the response to ICIs across multiple cancers, such as melanoma and NSCLC (6, 7). The common characteristic of tumors with high TMB is their high neoantigen load, which increases the visibility of the tumor to immune cells and activates a stronger antitumor response, potentiating their sensitivity to ICIs. The critical role of TMB is obvious within molecular subtypes of the same kind of tumor; for example, colorectal cancer patients with tumors demonstrating microsatellite instability or deficient mismatch repair and intact mismatch repair have a 40% and 0% response following anti-PD-1 therapy, respectively (47). However, there are exceptions, as low tumor mutational burdens and sensitivity to ICI therapies have been found, for instance, in metastatic renal cell carcinoma (48). Even so, as a general observation, low mutational load, and therefore low availability of neoantigens, is a main influencing factor for nonresponsiveness to ICIs. Herein, we conducted an integrative analysis with genomic and transcriptome data, which contributes to uncovering the underlying mechanism driving the establishment of immune clusters that correlate with the therapeutic effects of ICIs.

In a relatively large analysis of tumor datasets from 10 ICI trials, we estimated the relative abundance of tumor-infiltrating immune cell subsets in detail. The findings of this study may be reliable and generalizable. Nevertheless, limitations inevitably exist in our study. First, as a retrospective study, the proportions of immune cell subsets were computationally measured based on bulk transcriptomics from tumor tissues rather than quantified with experimental techniques such as flow cytometry, immunohistochemistry and newly developed single-cell RNA sequencing. Nevertheless, our findings are robust, as the findings of this study are in line with those of the publication regarding histologically characterized cohorts. Second, the diversity of ICB trials that utilized different checkpoint blockade agents, as well as different combinations collected in the analysis increases the generalizability but limit the specificity of our findings. Third, the findings reported here require experimental validation.

In conclusion, our study indicates that higher MHC scores, CYT scores and IFN-γ pathway activity are correlated with good prognosis following ICI therapy in melanoma. Additionally, we revealed that the fractions of CD8^+^ T cells, Tfh cells, activated memory CD4^+^ T cells and M1 macrophages were associated with a favorable response, PFS and OS after ICI therapy in melanoma and urothelial cancer. Moreover, resting memory CD4^+^ T cells were correlated with poor clinical outcomes. An immune cluster characterized by high levels of CD8^+^ T cells, Tfh cells, and activated memory CD4^+^ T cells and low levels of resting memory CD4^+^ T cells was identified and associated with high TMB/TNB and outperformed prognosis compared with the other immune clusters. These findings contribute to a deeper understanding of the tumor immune microenvironment for effective ICI therapy, improve the ability to predict and guide immunotherapeutic responsiveness and pave the way for drug combination strategies.

## Data Availability Statement

The original contributions presented in the study are included in the article/**Supplementary Material**. Further inquiries can be directed to the corresponding authors.

## Author Contributions

RL contributed to the study design, performed statistical analysis, interpretation and drafted the manuscript. RL approved the final version of the manuscript. All authors contributed to the article and approved the submitted version.

## Funding

This study was supported by the National Scientific Foundation of China (No. 31801121, 81974512, 81874329, 82073945), National Natural Science Foundation of Hunan (No. 2020JJ5879), Scientific Research Project of Hunan Provincial Health Commission (B2013-097) and Science and Technology Innovation Program of Hunan Province (2018SK50907).

## Conflict of Interest

The authors declare that the research was conducted in the absence of any commercial or financial relationships that could be construed as a potential conflict of interest.

## Abbreviations

ICI, immune checkpoint inhibitor; OS, overall survival; PFS, Progression-free survival; CIBERSORT, Cell type identification by estimating relative subset of known RNA transcripts; Tfh cells, T follicular helper cells; CTLA-4, cytotoxic T lymphocyte-associated protein 4; PD-1, programmed cell death 1; PD-L1, programmed cell death-Ligand 1; NSCLC, non-small cell lung cancer; FDA, Food and Drug Administration; IFN-γ, Interferon-γ; TME, tumor microenvironment; IL-10, interleukin-10; TGF-b, transforming growth factor-b; NK, natural killer; TPM, transcripts per kilobase million; ESTIMATE, Estimation of STromal and Immune cells in MAlignant Tumor tissues using Expression data; CYT, cytolytic activity; CR,complete response; PR, partial response; SD, stable disease; PD, progression of disease; OS, overall survival; PFS, progression-free survival; SqCLC, squamous cell carcinomas of the lung; ccRCC, clear cell renal cell carcinoma; HR, hazard ratio; CI, confidence interval; OR, odds ratio; Tfh, T follicular helper; TMB, tumour burden mutation; TNB, tumour neoantigen burden; SD, standard deviation; Tregs, T cells regulatory.
