# Supplementary material for: Influence of Tumor Immune Infiltration on Immune Checkpoint Inhibitor Therapeutic Efficacy: A Computational Retrospective Study
Source: Front Immunol. 2021 Jun 17;12:685370. doi: 10.3389/fimmu.2021.685370 (PMC8248490; doi:10.3389/fimmu.2021.685370)
Supplement: Supplementary file 2 [file DataSheet_2.docx]

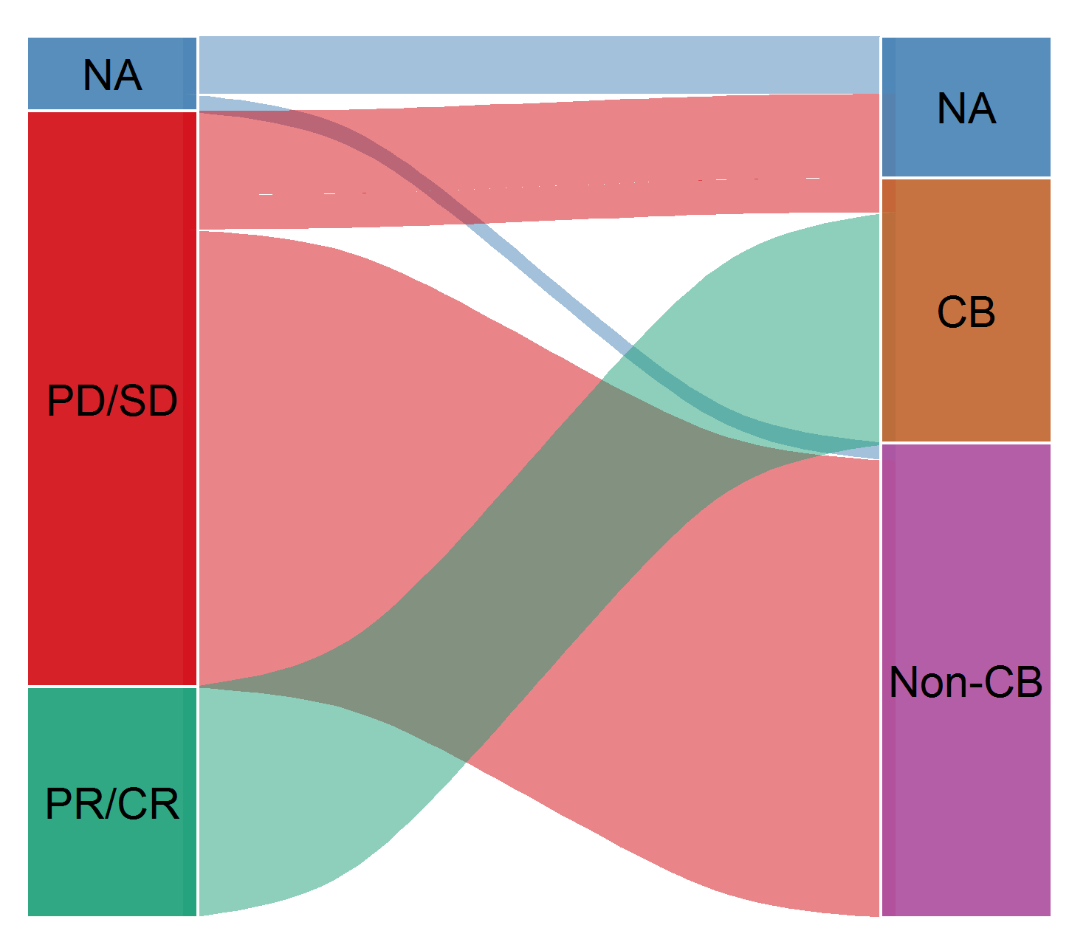


**Supplementary Figure 1**. Alluvial diagram for response to ICI therapy and clinical benefit.

CR: complete response; PR: partial response; SD: stable disease; PD: progressive disease; CB: clinical benefit.


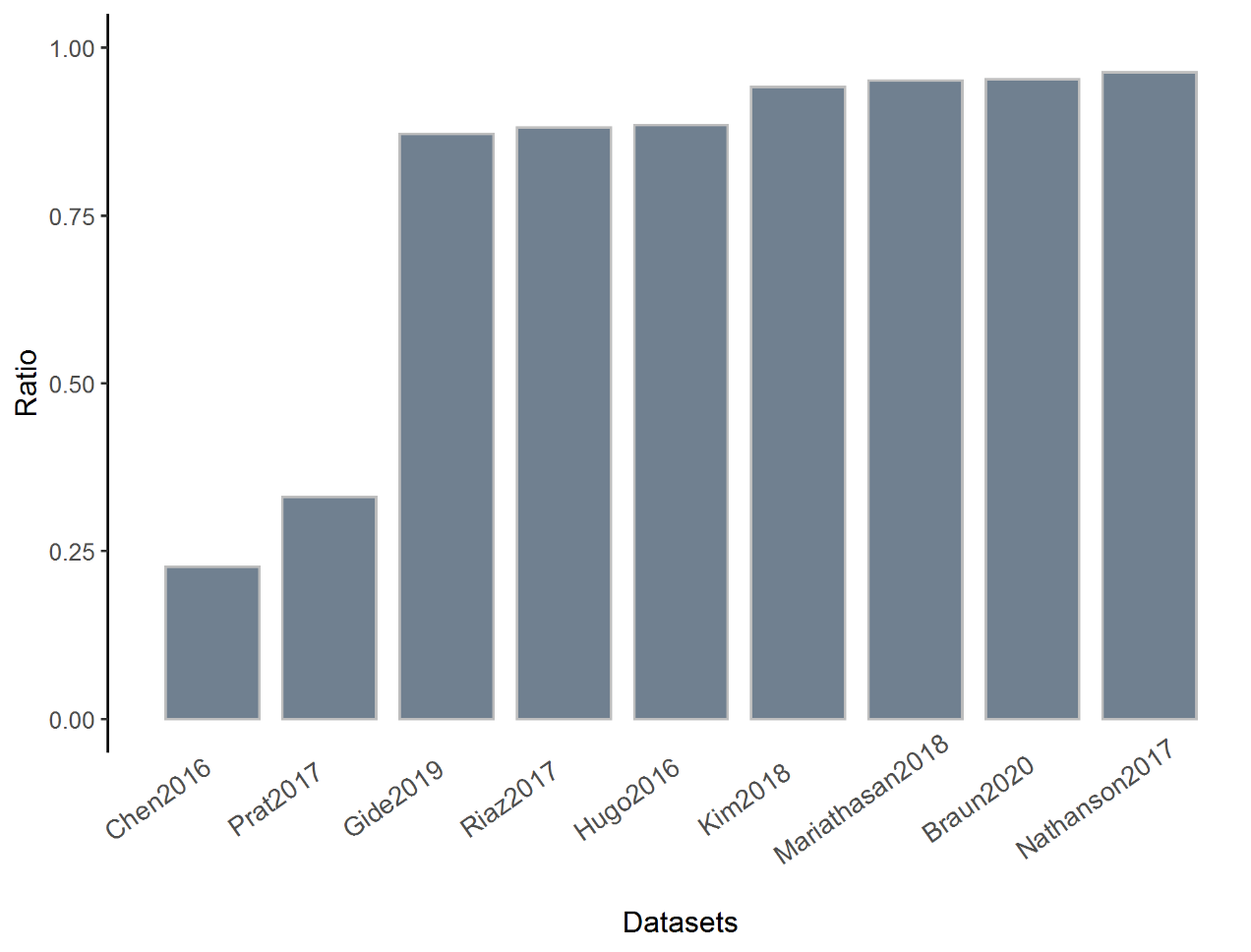


**Supplementary figure 2**: Bar plot of the proportion of the LM22 547 signature matrix genes available by datasets.

**
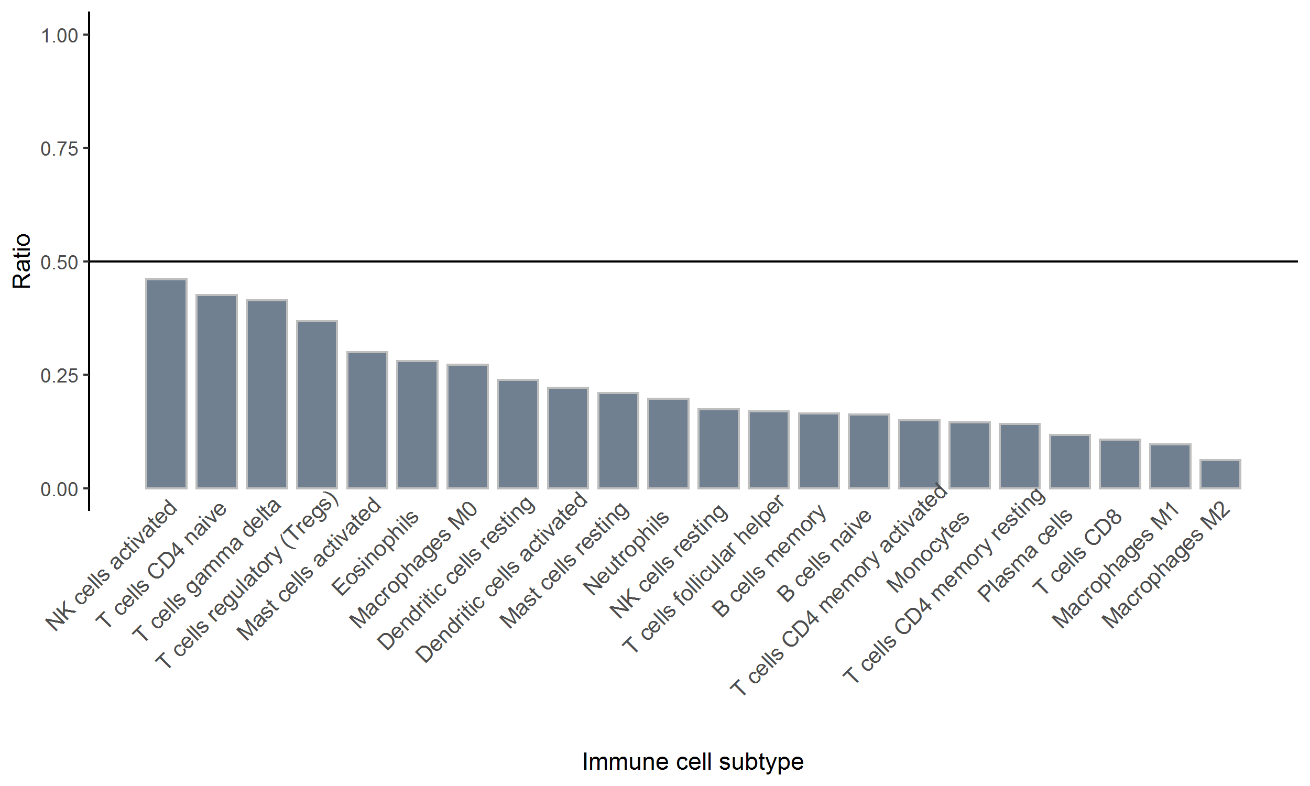
**

**Supplementary figure 3**: Bar plot of the overall proportion of the samples have no relative infiltration of individual immune cell subsets in the whole dataset.

**
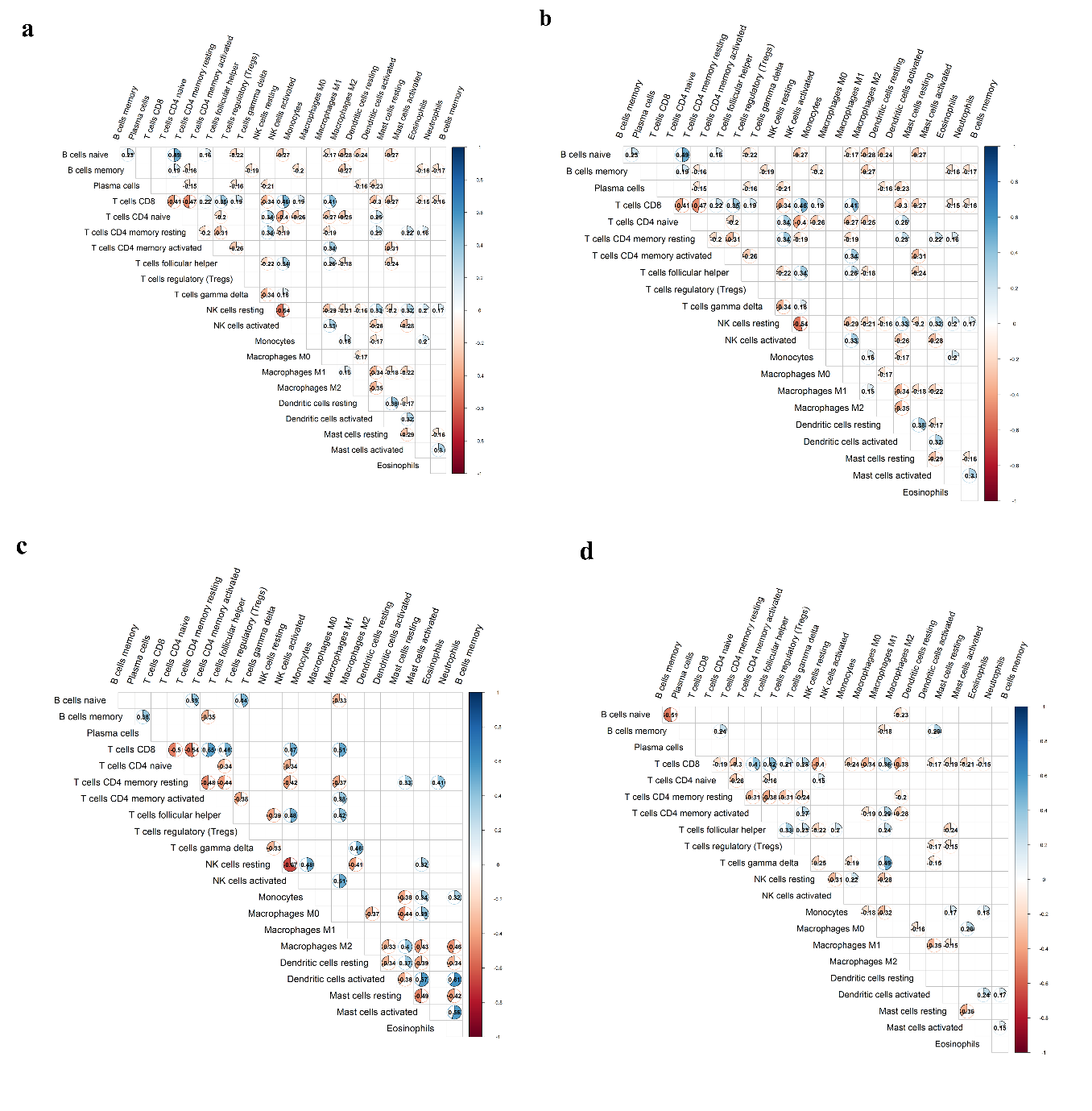
**

**Supplementary figure 4**. Correlation matrix of all 22 immune cell proportions in the melanoma (a), urothelial cancer (b), gastric cancer (c) and ccRCC (d) cohorts.

**
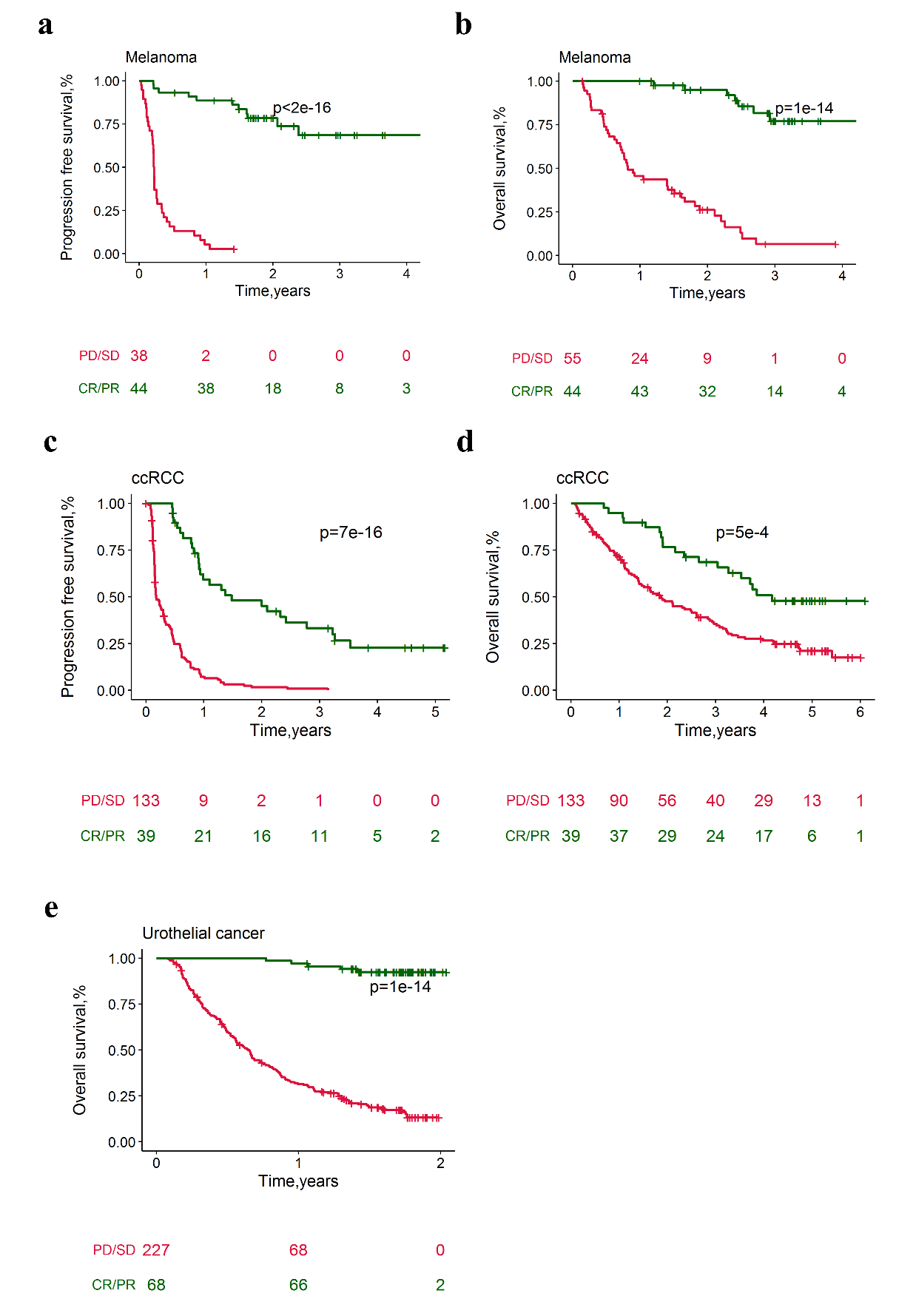
**

**Supplementary Figure 5.** Survival plots for overall survival and progression-free survival by response for melanoma (a-b), ccRCC (c-d) and urothelial cancer (e). P-values from log-rank tests are depicted.

**
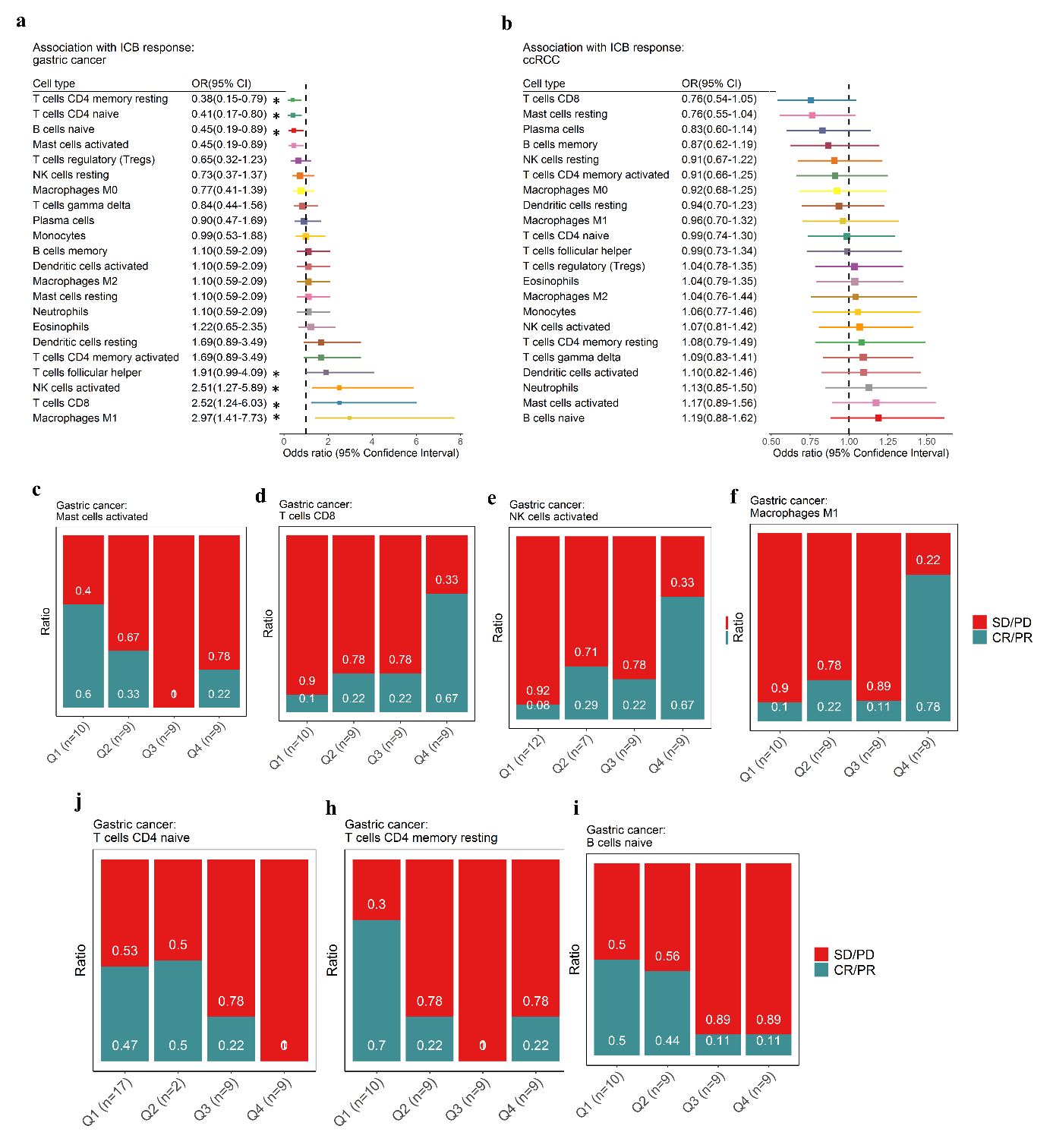
**

**Supplementary figure 6. Associations between ICI response and tumour-infiltrating immune cell subtypes.** Forest plot demonstrating ORs (boxes) and 95% CIs (horizontal lines) for the association with response to ICI therapy in gastric cancer (a) and ccRCC (b). The size of the box is negatively proportional to the standard error of the OR. * denote ORs with a p-value < 0.05. Spine plots demonstrating the distribution of response rates within quartiles of immune cell subsets (c-m). OR: odds ratio. CI: confidence interval.

**
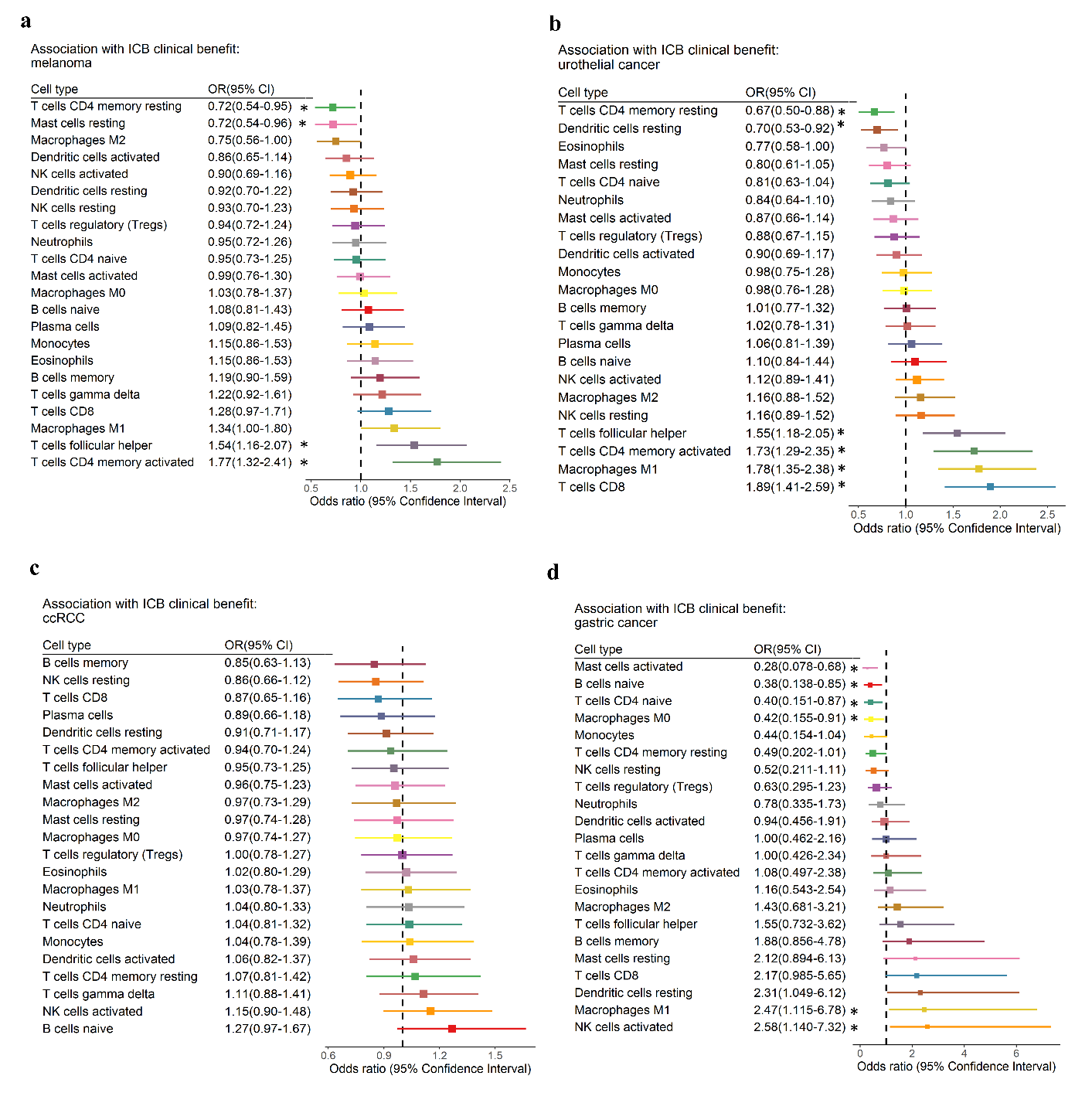
**

**Supplementary figure 7. Associations between clinical benefit and immune cells.** Unadjusted ORs (boxes) and 95% confidence intervals (horizontal lines) for the association are shown. Box size is inversely proportional to the standard error of OR. * denote a p-value < 0.05.

**
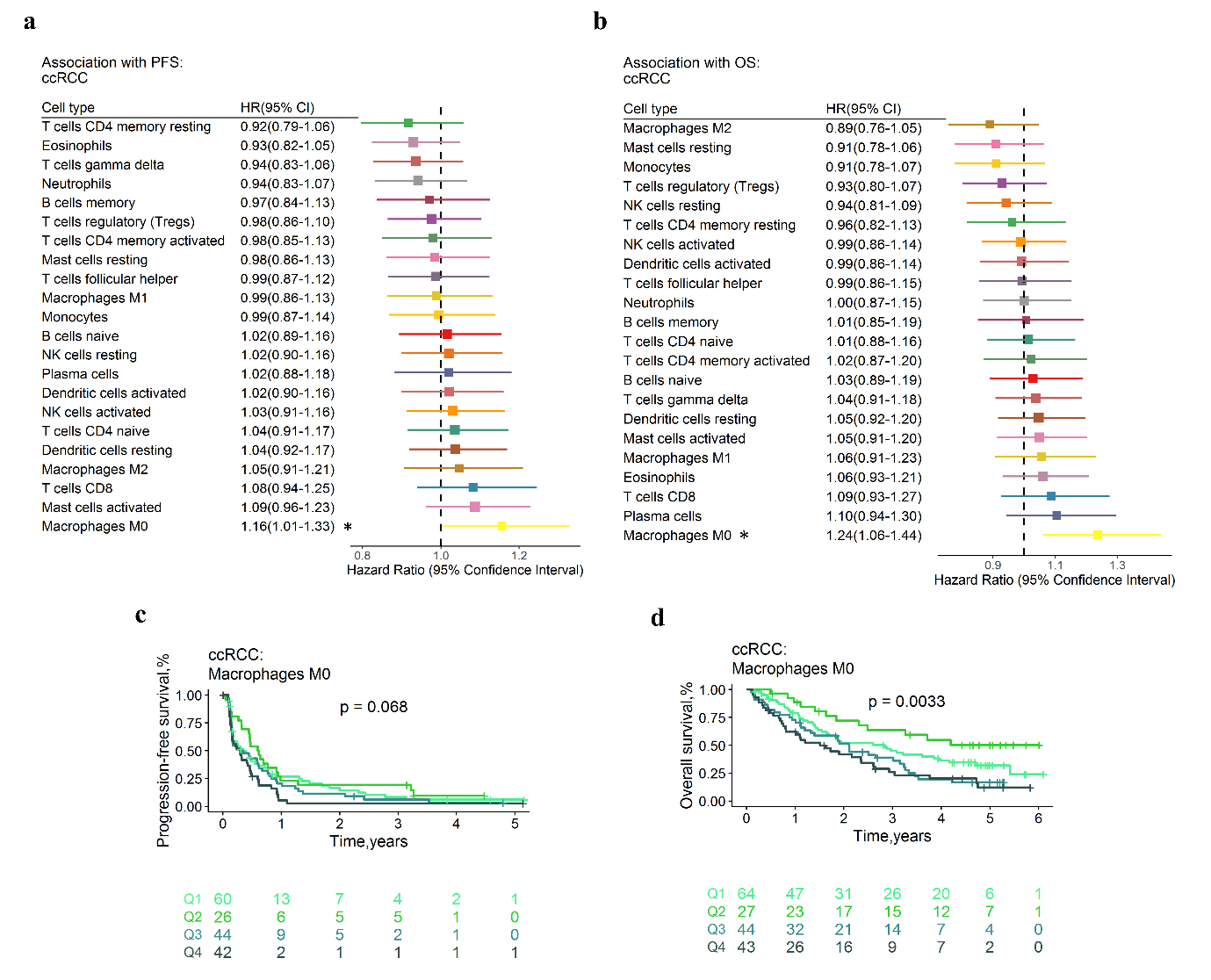
**

**Supplementary figure 8. Associations between immune cells and survival for ccRCC.** Unadjusted HRs (boxes) and 95% confidence intervals (horizontal lines) for the association were shown. Box size is inversely proportional to the standard error of HR. * indicates a p-value < 0.05. In the survival curves, immune cell subsets are stratified as quartiles (c–f). P-values from log-rank tests are shown. HR, hazard ratio.

**
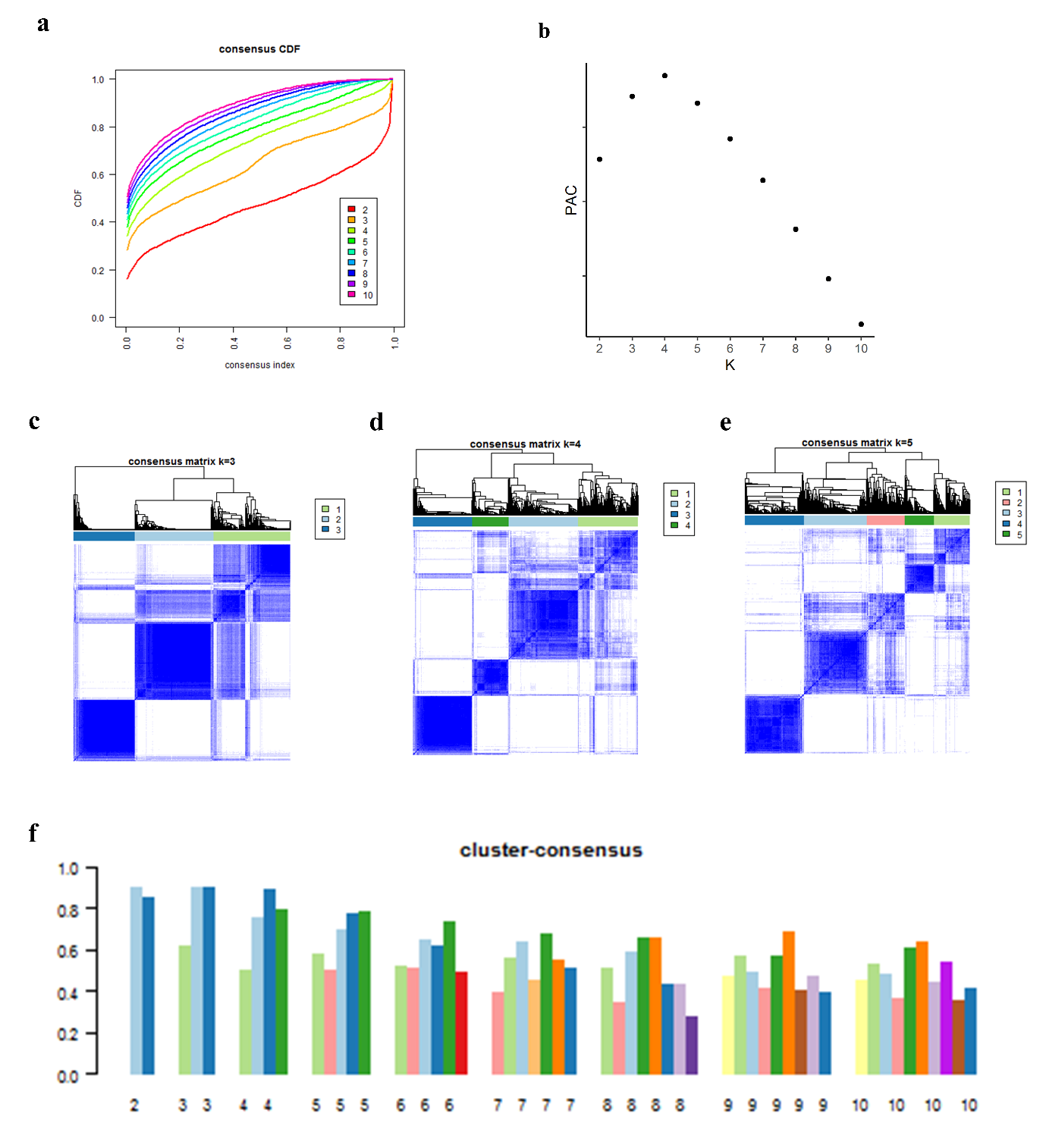
**

**Supplementary figure 9**. The cumulative distribution function (a) demonstrated that low k values at 3,4,5 underfit the dataset whereas k=8,9,10 overfits the data. To discern whether k=3 or k=4 or k=5 better fit our dataset, we calculated the proportion of ambiguous clusters (PAC) values (b). Both the consensus matrix (c-e) and consensus cluster index (number of times cluster stays consistent; f) also supported that k=4 was more stable than k=3 or k=5.


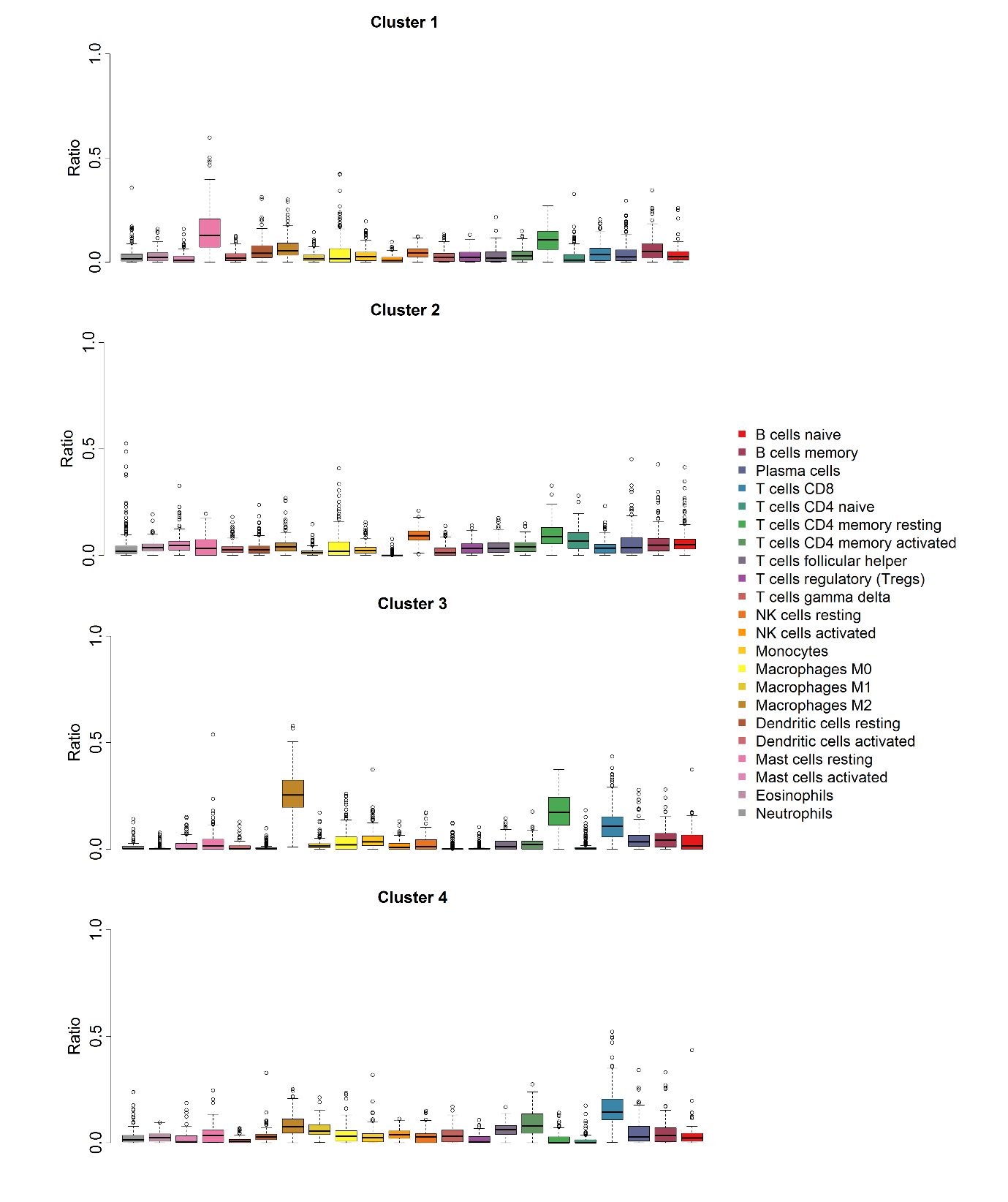


**Supplementary figure 10.** Box plots depicting the distribution of each immune cell type across four immune cluster.
